# 

**Published:** 1893-07

**Authors:** 


### CONTENTS—JULY, 1893.—VOL. IX., NO. 1.

ORIGINAL COMMUNICATIONS—
The Mary Newberry Trial. D. R. Wallace, M. D., LL. D., Waco . . 1
Spina Bifida. Report of a Case. Jas. Kennedy, M. D., San Antonio 16
Pelvic Inflammation in the Female. W. C. Fisher. M. D., Galveston . 20
ABSTRACTS OF CURRENT MEDICAL LITERATURE—
Department of Therapeutics. Dr. David Cerna, Galveston . . . . . 23
Department of Obstetrics and Gynecology. Prof. Keiller . . . . . . 27
SOCIETY NOTES—
Austin District Medical Society . . . . . . . . . . . . . . . . . . . 30
Johnson County Medical Society . . . . . . . . . . . . . . . . . . 30
EDITORIAL—
Special Provision for Epilepsy . . . . . . . . . . . . . . . . . . . 31
Newspapers and the Doctors . . . . . . . . . . . . . . . . . . . 32
Pathological Museum University of Texas . . . . . . . . . . . . . 34
Deliver us from our Friends . . . . . . . . . . . . . . . . . . . . 35
Blood on the Medical Moon . . . . . . . . . . . . . . . . . . . 36
Charity Hospital Training School for Nurses . . . . . . . . . . . . 36
Dr. Richmond B. McKinney . . . . . . . . . . . . . . . . . . . . 36
MEDICAL NEWS AND MISCELLANY—
Numerous paragraphs of general interest to the profession . . . . . . 37
BOOK NOTICES—
Psychopathia Sexualis—41. Elements Human Psysiology—41. Cholera—42. Guide to Disection—43. Life of D. Hays Agnew—44.
PUBLISHERS' NOTES . . . . . . . . . . . . . . . . . . . . . . 46

